# Generalizability of developmental EEG: Demographic reporting, representation, and sample size

**DOI:** 10.1016/j.dcn.2025.101567

**Published:** 2025-05-13

**Authors:** Santiago Morales, Lauren Oh, Kylie Cox, Ramiro Rodriguez-Sanchez, Gina Nadaya, George A. Buzzell, Sonya V. Troller-Renfree

**Affiliations:** a Department of Psychology, University of Southern California, Los Angeles, CA, USA; b Department of Psychology, California State University Dominguez Hills, CA, USA; c Department of Psychology, Florida International University, Miami, FL, USA; d Center for Children and Families, Florida International University, Miami, FL, USA; e Department of Human Development, Teachers College, Columbia University, New York, NY, USA

**Keywords:** EEG, ERP, Diversity, Representation, Demographics, Replicability

## Abstract

Electroencephalography (EEG) is one of the main neuroscientific measures used with infants and children to identify potential biomarkers of cognitive and social developmental processes. Given the implications of developmental EEG research within policy, clinical, and educational domains, it is important to ensure that reported results are generalizable and reproducible. In this review, to provide an initial assessment of previous and current practices regarding participant recruitment (sample size and representation) and demographic reporting, we carried out a systematic review of six notable journals for publishing pediatric EEG studies between 2011 and 2023. We identified 700 articles reporting on pediatric EEG. We found that most studies did not provide complete reporting of basic demographic information (e.g., race, ethnicity, socioeconomic status, geographical location). This trend persisted across years of publication, suggesting continued underreporting. However, the reporting of demographic information differed between journals, suggesting solutions for improving reporting practices. Our review also indicated that samples were of modest sample size (Median = 51) and consisted of mostly White participants (78 %) from North America and Western Europe (85 %). Our discussion emphasizes the need for larger, more diverse samples and greater transparency in developmental EEG studies, while providing recommendations to address barriers to representation and reproducibility.

## 1 Introduction

In the 1920s, electroencephalography (EEG) was utilized for the first time to record activity from the human brain (Berger, 1929). Over a century later, EEG continues to be an instrumental neuroimaging tool, allowing us to obtain insight into the neural mechanisms underlying many psychological, cognitive, and affective processes (Mushtaq et al., 2024). EEG has become a particularly useful method in developmental neuroscience research, given its cost-effectiveness, robustness to motion, and high temporal resolution (Buzzell et al., 2023). This has led to important discoveries about the development of cognitive and affective processes, and has provided unique measures of individual differences across development (Barry-Anwar et al., 2020, Bell and Cuevas, 2012, Morales and Fox, 2019, Saby and Marshall, 2012). In addition to its contributions to the fields of neuroscience and developmental psychology, EEG has been used for informing policy change (Nelson et al., 2014, Troller-Renfree et al., 2022), clinical screening and treatment (McPartland et al., 2020), and educational practice (Davidesco et al., 2023, Thomas et al., 2019). As developmental EEG research becomes increasingly influential in the study of developmental processes and as it generates more implications in policy, clinical, and educational settings, it is important to ensure such work is generalizable and reproducible. To encourage more generalizable and reproducible findings, the current report focuses on the role of demographic reporting, representation, and sample size.

## 2 Reporting and representation of participants’ demographic information

One important factor that determines the generalizability and reproducibility of developmental EEG research is the characteristics of the samples utilized. It is well-established that most psychological research is conducted on White, Educated, Industrialized, Rich, and Democratic (WEIRD) samples, which are not representative of the global population (Henrich et al., 2010). This is also true of developmental neuroscience, including EEG research. This is problematic because it questions the validity, replicability, and generalizability of current findings beyond the selective samples on which they are based. It has been extensively documented that there are important differences in EEG measures based on different demographic characteristics of participants. For instance, differences in EEG findings have been reported based on sex/gender (Hanlon et al., 1999), race and ethnicity (Lahat et al., 2010, Rapp et al., 2021, Rapp et al., 2021), SES (Conejero et al., 2018, Jensen et al., 2021, Sandre et al., 2024, Tomalski et al., 2013; X. Xu et al., 2024), and place of recruitment (Alahmadi et al., 2016).

Moreover, recent evidence suggests that some findings from EEG studies differ based on the demographic characteristics of the participants and do not always generalize to other populations. For instance, Jensen et al. (2021) found the opposite relation between SES and EEG power in children in Bangladesh to what is often found in WEIRD samples (e.g., (Tomalski et al., 2013). Another example is a study by Rapp et al. (2021), in which neural responses to rejection were associated with social anxiety among non-Latinx White and Asian American adolescents, but no relation was present for Latinx adolescents. Similarly, another study by the same group found a link between values of collectivism and heightened neural responses to errors in a team context (i.e., the participant’s error was costly to their team), and these effects were most noticeable among Latinx participants (Rapp, Grammer, et al., 2021). Together, these studies illustrate important differences based on the demographic characteristics of participants, as well as how EEG measures relate to other outcomes, highlighting the importance of reporting these measures and including representative samples. At the same time, it is important to note that there are contexts in which asking about some demographic information is not appropriate due to cultural norms, historical reasons, and legal restrictions. For example, in some countries, collecting data on race and ethnicity is not be the norm or even legal due to past experiences with genocide and prosecution. Because of this, it has been recommended that researchers report the appropriate demographic characteristics of the sample to the extent that it is possible (Gatzke-Kopp et al., 2023).

It has been documented that in both neuroscience and developmental science, there is a lack of representation and reporting of basic demographic characteristics of the samples involved in studies. For the broader field of neuroscience, a recent systematic search by Goldfarb and Brown (2022) examined the demographic reporting of neuroimaging studies (EEG and fMRI) in 2019. The review found that only 4 % of the articles in top cognitive neuroscience journals reported the race or ethnicity of their participants. Importantly, of the reviewed studies involving EEG, none of them reported the racial and ethnic information of the participants and these results did not differ by journal (Goldfarb and Brown, 2022). Similarly, a recent review of the reporting and analysis of participant demographics in the journal *Psychophysiology* (one of the top outlets for EEG research) found that race or ethnicity was reported in only 17 % of the articles, and SES was almost never (∼2 %) reported, whereas, gender or sex were reported on almost every manuscript (Kissel and Friedman, 2023). Notably, these patterns of reporting were similar across the years examined (2010–2020). Although some missing demographic data is to be expected—given that some measures to describe participants’ communities of descent cannot be utilized in specific contexts due to cultural norms, historical reasons, and legal restrictions (e.g., race and ethnicity are predominantly utilized in the US)—these two reviews highlight there is a dearth in the reporting of major demographic variables in the top neuroimaging and psychophysiological journals. Importantly, these reviews examined journals that mostly publish research involving adults, leaving the patterns of reporting and representation in developmental neuroscience unexamined.

To our knowledge, no comprehensive reviews have been conducted involving the representation and reporting in the emerging field of developmental neuroscience. One commentary briefly mentioned that only ∼37 % of manuscripts published in 2020 in *Developmental Cognitive Neuroscience* (one of the top journals) reported on the race, ethnicity, and/or SES of study participants (Garcini et al., 2022). Although this has not been comprehensively examined in developmental neuroscience research, similar reviews have been conducted in developmental science more broadly. For instance, Singh et al. (2023) investigated the trends of socio-demographic data in infant research in articles from top developmental journals published from 2011 to 2022. The review found that participant demographic information was largely excluded from the literature, such that most studies (53 %) did not report the race or ethnicity of the participants. Moreover, even when the demographic data was reported, samples tended to consist primarily of White infants and families from Western Europe and North America (84 %). Finally, the trends in reporting were similar across the years examined (2011–2022), highlighting the lack of progress in reporting basic demographic information in infant research.

### 2.1 Sample size of studies

In addition to the sample composition and its reporting, another important factor influencing the reproducibility, representation, and generalizability of studies is the sample size. Large samples reduce the risk that results reflect idiosyncratic characteristics of a small group and increase confidence that the findings will generalize to the general population. Moreover, larger samples increase the likelihood that the sample has sufficient variation and is representative across several demographic factors. Also, sample size determines the statistical power of a study, such that small samples can lead to underpowered studies, which make it less likely to detect smaller effect sizes as well as lead to unreliable effect sizes (Loken and Gelman, 2017, Oakes, 2017). In this way, small sample sizes can undermine the replicability and generalizability of scientific research, which may be a particularly salient concern for developmental neuroscience given costs and participant compliance.

Existing reviews have documented the reliance of small samples in neuroscience research in general (Button et al., 2013). Although there has been sizable interest in the reproducibility and power of fMRI data (e.g., Szucs and Ioannidis, 2020), less interest has been given to EEG data. A recent review of the EEG literature in the top journals for Event-Related Potential (ERP) research documented that small samples are also prevalent in EEG research with adults (Clayson et al., 2019). The review found that, across 150 studies published from 2011 to 2017, the average sample size per article was 29 (median = 22), and the average sample size per group was 21 (median 18). This suggests that studies published in these high-impact ERP journals are likely not representative of the general population and only powered to detect large effect sizes. Again, we are not aware of similar reviews for developmental neuroscience, but this might be a particularly salient concern for developmental EEG studies given the increased noise from motor artifact and increased data loss compared to adult studies. However, small sample sizes are also common in research conducted on infants and young children, given the difficulties associated with recruiting participants, collecting data, and training experimenters. Oakes (2017) conducted a review of the literature on infant-looking times and found that studies tend to consist of small sample sizes, with most studies having only 11–24 infants per cell and few having more than 25 infants per group. This suggests that infant-looking studies are predominantly powered to detect large effect sizes. Interestingly, it appears as though sample sizes were largely determined by convention and rule of thumb instead of through power analyses (Oakes, 2017), or careful sampling strategies to represent specific communities. Together, these reviews highlight the need to examine sample sizes, in tandem with demographic composition, in developmental neuroscience and whether trends in sample sizes have changed over time.

### 2.2 Current study

The existing evidence suggests that there is an important lack of reporting of basic demographic information in the top neuroscience and developmental journals. Moreover, previous reviews highlight that most EEG studies with adults and developmental research with infants rely on small sample sizes comprised of mostly White participants from Western Europe and North America. However, no reviews have been conducted for developmental neuroscience and less so regarding developmental EEG. Developmental neuroscience may particularly suffer from a lack of representation and small sample sizes given the costs of the equipment and the difficulty collecting such data in special populations such as infants and children. Moreover, EEG has unique methodological issues that potentially impact representation. This is because EEG requires contact between the electrode and scalp to measure brain activity, which is more easily accomplished with thin, straight hair, making it more difficult for specific hair types or hairstyles commonly worn by some racial and ethnic groups (Adams et al., 2024, Bradford et al., 2024, Choy et al., 2022, Webb et al., 2022). Because of these factors, along with the increased relevance of developmental EEG in policy, clinical, and educational settings, it is particularly important to document both the reporting practices, as well as the sample composition of developmental EEG studies. In the current study, we conducted a systematic review of six notable journals publishing pediatric EEG to study developmental psychology to provide an initial assessment of previous and current practices involving participant recruitment and documentation. To identify potential solutions and historical trends, we also examined differences between journals and changes across the last 12 years (2011–2023).

## 3 Methods

We first identified the developmental journals with the most published EEG manuscripts that included children between 2011 and 2021 (when the initial search was conducted; subsequent coding of articles was later updated to include studies published between 2011 and 2023). Using a similar approach to Clayson et al. (2019), we utilized PubMed to identify all articles published that included relevant keywords in the title, abstract, or manuscript text. Examples of these keywords included “EEG,” “ERP,” “infant,” “child,” “adolescent,” and “pediatric” (a complete list of the keywords and search terms can be found in the supplement). Given our focus on the use of EEG to study developmental psychology, we excluded clinical journals where EEG is primarily utilized to diagnose seizures (e.g., *Epilepsia* or *Epilepsy and Behavior*). After excluding these clinical journals (e.g., journals focused on epilepsy or neurology), we selected the journals with the most published papers on three categories to capture a wide variety of journals publishing developmental EEG studies. We selected two developmental neuroscience journals (*Developmental Cognitive Neuroscience* and *Developmental Psychobiology*), two general developmental journals (*Developmental Science* and *Child Development*), and two general journals (*PLOS One* and *Scientific Reports*). We did not select general neuroscience journals (e.g., *NeuroImage* or *Psychophysiology*) because they have recently been the target of similar analyses (e.g., Goldfarb and Brown, 2022; Kissel and Friedman, 2023). All articles published in these six journals and matching our keywords (1072 articles) were identified using PubMed and uploaded for screening and review to Covidence (Veritas Health Innovation, 2023). Covidence is a web-based collaboration software platform that streamlines the production of systematic and other literature reviews.

**Fig. 1:**
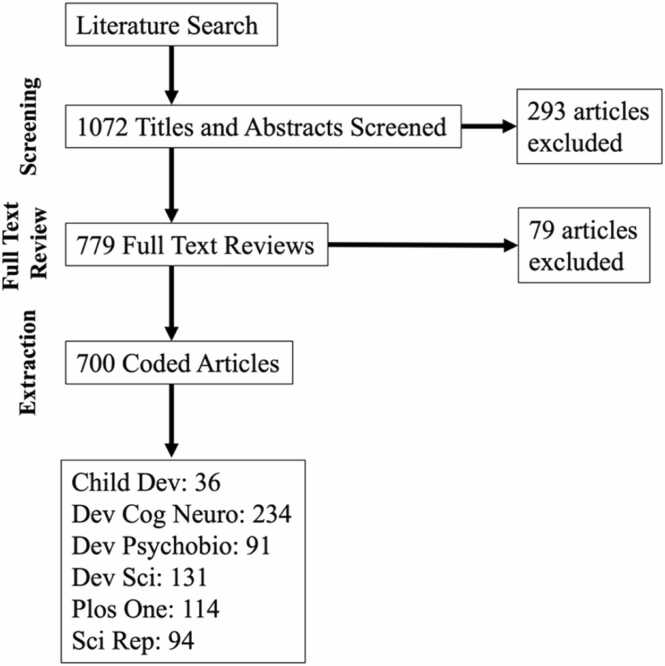
Flowchart of article identification, screening, and coding.

We screened titles and abstracts of 1072 studies to identify 700 empirical articles reporting on EEG from children (newborns to 17-year-olds). We excluded review articles, commentaries, meta-analyses, animal studies, studies utilizing only other neuroimaging methods (e.g., fNIRS, fMRI, MRI), stimulation procedures (e.g., TMS), physiological measures (e.g., EMG) or behavioral methods, studies only including adults, or collecting EEG but using for clinical purposes rather than research (e.g., aEEG or iEEG). Fig. 1 shows the process of article selection as a PRISMA flow diagram, as well as the final number of articles identified for each journal.

For the selected articles, we examined the full article and coded the following characteristics based on the information provided by the authors: Sample Size, Race, Ethnicity, Sex/Gender, Socioeconomic Status, and Location/Country of recruitment. Note that for the purposes of this review, we coded reporting of either sex or gender as a single variable. In contrast, although race and ethnicity are often reported together, we coded and reported separately. Sensitivity analyses show the same pattern of results if examining race and ethnicity as one variable (see Results). Moreover, because race and ethnicity are most used in the US, we conducted sensitivity analyses limiting the reporting analyses to studies conducted in the US (see Results). Finally, we acknowledge that these measures are imperfect measures to evaluate individuals’ sociocultural and demographic backgrounds, encompassing large, heterogeneous groups. However, building on similar efforts, we utilize them as our main measures as they are widely used by researchers to communicate sample composition (Goldfarb and Brown, 2022, Singh et al., 2023).

The demographic information was coded for all participants in the study who had EEG data collected. For some articles, this also included adult participants. For example, some studies compared children and adults, or also collected EEG in caregivers (e.g., hyperscanning). Four coders coded the data with adequate reliability for most codes (Kappa: Mean =.88; Range =.79–.95; Percent agreement: Mean = 93.7 %; Range = 90.3 %-96.6 %). For these codes, reliability was determined based on double coding ∼25 % of the data. The only code that did not display adequate reliability in the initial ∼13 % of the data was the location/country of recruitment code (Kappa =.58; Percent agreement = 69.7 %). As such, we decided to double-code all manuscripts for the location/country of recruitment code, which resulted in good reliability (Kappa =.83; Percent Agreement = 87.7 %), and any discrepancies were resolved by discussion resulting in complete coder agreement.

### 3.1 Data analysis

Our analyses are divided into four sections. First, we summarize the overall rates of reporting for each demographic category. To examine differences between journals, we conducted chi-square analyses. To examine changes across years of publication and identify trends in reporting, we correlated rates of reporting with the year of publication. A significant correlation would indicate significant increases in reporting. Note that we were not able to examine an interaction between the journal and year of publication because the cell sizes were too small to provide robust estimates.

Second, we examined the demographic composition of the studies reporting participant demographics. For this, we extracted the total number of participants and/or the proportion of participants who fell into different racial, ethnic, and sex/gender categories. Even when studies reported the demographic categories of interest, it was not in a standardized manner. For example, some studies only reported proportions, while others reported raw numbers. If a study only reported proportions, we utilized their reported sample size to estimate the number of participants and vice versa. Finally, for our main analysis, we report the mean and median number and proportion of each demographic category within each study, providing a description of the representation of existing developmental EEG studies. In addition, we provide the total number and proportions of participants within each category across all studies, which provides a sense of the overall representation of developmental EEG when combining all studies.

Third, when examining the geographical location of recruitment and data collection, we focused on the country in which the data collection took place. Because of the lack of reporting (see below), we also decided to infer where the data were collected by using author affiliations and the IRB that approved the study, which are strong (although imperfect) indicators of where the study took place. To examine differences between journals and year of publication, we conducted similar analyses, as described above.

Fourth, when analyzing sample sizes, we summed across studies that utilized several groups or substudies (in the case of multi-study manuscripts). When examining patterns in sample sizes, we employed non-parametric measures because sample sizes were not normally distributed. When examining differences between journals, we utilized the Kruskal-Wallis test, which is a non-parametric alternative to an ANOVA. To examine changes across years of publication and identify trends in reporting, we correlated sample sizes with the year of publication using Spearman’s rank correlation. Note that we were not able to examine an interaction between the journal and year of publication because the cell sizes were too small to provide robust estimates. Finally, for power analyses, we utilized the *pwr* R package (Champely, 2020).

## 4 Results

### 4.1 Rates of reporting participant demographics

When examining the full text of each article, results revealed that most studies did not report basic demographic information across race, ethnicity, SES, and location/country where the study was carried out. Of the studies reviewed, only 28 % reported race, 16 % reported ethnicity,^1^ 32 % reported SES, and 89 % reported sex/gender. Similarly, when examining the geographical location of data collection, we observed that only 48 % of the studies reported the location (i.e., country) of recruitment.

**Fig. 2:**
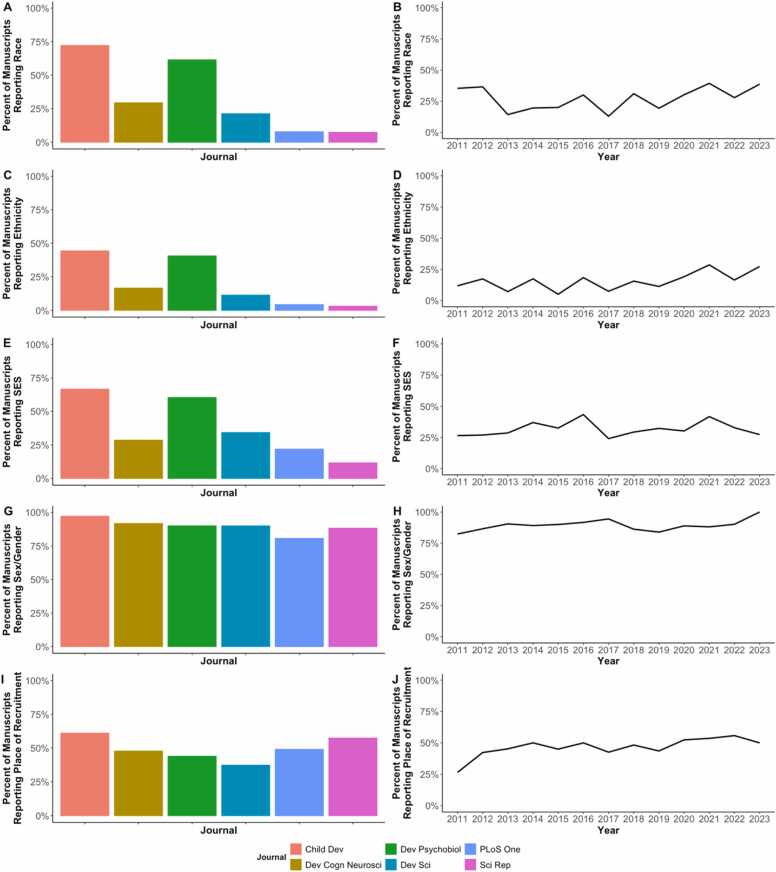
Proportion of manuscripts with reports of key demographic information by the journal (left column) and year of publication (right column). The demographic measures correspond to race (A & B), ethnicity (C & D), SES (E & F), sex/gender (G & H), and geographical location (I & J). Note that for geographical location, the rates indicate only the studies that reported data and not the inferred countries shown in Fig. 3.

As illustrated in Fig. 2, for the most part, these trends did not significantly change based on the year the study was published (between 2011 and 2023), such that the percentage of studies reporting these sample characteristics was not related to publication year (*r*’s < =.45, *p*’s > =.12). The only exceptions were the reporting of ethnicity (*r* = .56, *p* = .044) and place of recruitment (*r* = .71, *p* = .007), both of which increased in their report during recent years. However, it is worth noting that in 2023, ethnicity reporting was still under 30 %, and place of recruitment reporting was around 50 %.

The reporting of sample characteristics significantly differed between journals (χ^2^(5)’s > = 13.3, *p*’s < =.02). As shown in Fig. 2, in general, papers published in *Child Development* tended to have better rates of reporting. This difference was present across demographic characteristics, including those sample characteristics that were less commonly reported like ethnicity, as well as others more commonly reported like sex/gender.

Notably, the rates of reporting of race and ethnicity significantly differed by country in which the study took place (χ^2^(1) = 103.8, *p* < .001)–such that studies were more likely to report race and/or ethnicity if they took place in the US (n = 272), compared to other countries (n = 428). Nevertheless, even when solely considering studies from the US, only 51 % reported race and 32 % reported ethnicity.

### 4.2 Study composition for studies reporting participant demographics

**Race and Ethnicity:** For studies that reported any of the race or ethnicity demographics, we examined the composition of their samples. However, when studies did not provide numeric or proportion information (e.g., several studies only reported the composition of their sample descriptively as “predominantly White” or “most participants were White”), they were not included in this analysis. Table 1 includes the total number of participants and proportion for demographic category across all studies, as well as the median and mean proportion of each demographic category as reported within each study. As shown in Table 1, of the studies that reported race and ethnicity with enough detail (26 % of all studies; N = 182), samples consisted of primarily White participants. This pattern in the sample composition was consistent across measures, including the main results reported on the manuscript. One notable discrepancy is the proportion of Asian participants for the measures collapsed across all studies, compared to the measures within each study. This is because there was one large study composed of only Asian participants, which accounted for most Asian participants; thus, this is reflected in the total of participants but not in the measures within samples. It is important to note that these estimates only include the articles that reported race and ethnicity quantitatively, so it is likely overestimating the true diversity in the samples of pediatric EEG, as studies with less diversity are possibly more likely to not report race/ethnicity information.

**Table 1:** Proportion of participants with demographic information reported across journals for the subset of studies that reported relevant demographics.

**Demographic** **Variable**	**Total Number of Participants**	**Proportion of Total Number of Participants (%)**	**Median Proportion of Each Study (%)**	**Mean Proportion of Each Study (%)**
White	16523	54.92	78.17	72.98
Asian	7080	23.53	3.90	14.98
Black	2607	8.66	9.25	14.10
Mixed	1643	5.46	9.38	12.80
Hispanic/Latino/a/x	1361	4.52	6.63	12.27
Not Reported	700	2.33	6.70	9.34
American Indian/Alaska Native	135	0.45	1.60	2.27
Native Hawaiian/Pacific Islander	38	0.13	2.00	2.56
Male	24926	51.03	51.26	52.81
Female	23916	48.97	48.50	47.09

Note: This only includes a subset of studies that reported the relevant demographics quantitatively (26 % for Race and Ethnicity, and 89 % for Sex/Gender). For studies that only reported percentages of participants within each demographic category, we computed the number of participants within each category by multiplying percentages by the provided sample size, and vice versa to obtain the number estimates presented in the supplement. Derived sample size numbers that were not exact were rounded to the nearest number. The proportions do not add perfectly to 100 % because what different authors included in their estimates varied by study (e.g., reporting ethnicity separately from race), and the averages were additionally influenced by outliers.

**Fig. 3:**
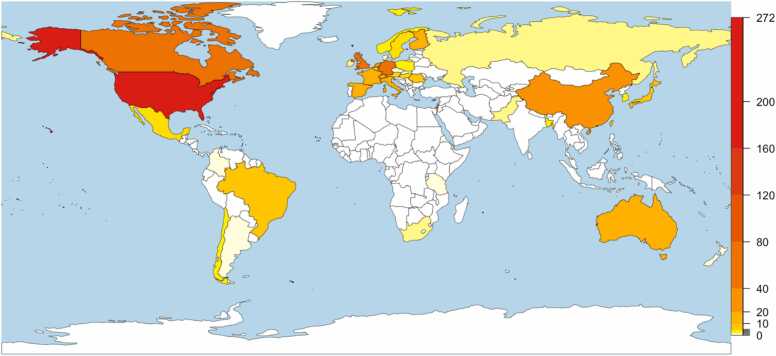
A geographical illustration of the number of studies by inferred country. The plot was created with the *rworldmap* R package (South, 2011).

**Sex/Gender:** In contrast to the rates of race and ethnicity, for the studies that reported sex/gender quantitatively (89 % of all studies; N = 622), the rates of males and females were relatively equal across studies. These patterns were also consistent when examining sample composition collapsed across studies, providing an overall estimate of participant representations across all reviewed studies (Table 1).

**Geographical Location of Recruitment:** When examining the place of recruitment and data collection, we focused on the country in which the data collection took place. Of those that indicated the place of recruitment (48 % of all studies), the vast majority were from North America (42 %) and Western Europe (40 %). Because of the lack of reporting, we decided to infer where the data were collected by first using the IRB that approved the study and if not available, used the first author’s affiliation, which are strong (although imperfect) indicators of where the study took place. After inferring the location of recruitment, we observed a similar pattern of results. As shown in Fig. 3, most studies come from North America (45 %) and Western Europe (40 %), while very few studies come from Latin America (3 %), South Asia (2 %), and an especially small portion from Africa (<1 %). The countries with the most studies were the US (38 %), Germany (10 %), UK (10 %), and Canada (8 %). In general, the global south seems to be particularly underrepresented in developmental EEG research.

**Fig. 4:**
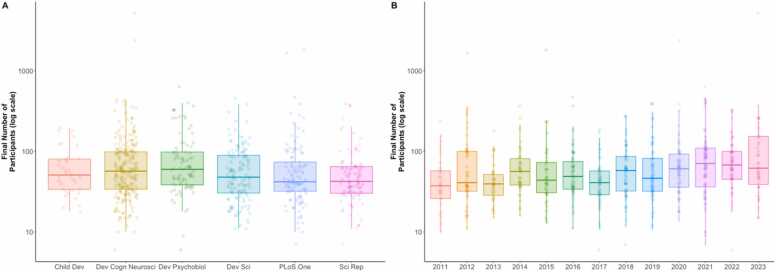
Box plots of sample size by journal or year of publication.

**Sample Size:** When examining patterns in sample sizes, we employed non-parametric measures because sample sizes were not normally distributed. We found that the median total sample size was 66 participants. This number reflects the number of participants before removing participants (e.g., due to lack of artifact-free trials) for many studies. When examining the number of participants included in reported analyses, we found a median final sample size of 51 participants. This suggests that most studies were adequately powered (.80) to detect large to medium effect sizes for between-subject analyses (*d* ≥.80, *r* ≥ .38) and small to medium effect size for within-subject analyses (*d* ≥.58, assuming a correlation of *r* = .5 between measures), given that within-subject analyses generally have more power to detect smaller effects. However, as illustrated in Fig. 4, there was considerable variation in sample sizes, ranging from 6 to 5207 participants. Moreover, as shown in Fig. 4A, sample sizes differed by journal (χ^2^(5) = 14.37, *p* = .013). Post-hoc analyses revealed that Developmental Psychobiology had larger samples than PLOS One, Scientific Reports, and Developmental Science (*W*’s > = 6987, *p*’s < =.029). Similarly, Developmental Cognitive Neuroscience tended to have larger samples than PLOS One and Scientific Reports (*W* = 15026, *p*’s < .039). Finally, as shown in Fig. 4B, when examining the year of publication, we found that sample sizes tended to increase in recent years (Spearman *ρ* =.20, *p* < .001) from a median sample size of 38 in 2011 to 62 in 2023.

## 5 Discussion

The current review provides a summative overview of reporting and recruitment practices of developmental EEG studies. We examined the reporting and sample composition of all pediatric EEG manuscripts published in six journals that are notable outlets for developmental EEG between the years 2011 and 2023. Findings highlight issues with underreporting of key demographics as well as a lack of representation, especially global representation. These patterns raise important questions regarding bias in and generalizability of existing developmental EEG studies. Below, we outline recommendations and potential solutions to improve the reporting and transparency of developmental EEG, as well as increase the representation of developmental EEG.

### 5.1 Recommendations for improving the reporting of demographic information

In line with the existing reviews of both neuroscience and developmental fields, there is considerable under-reporting of key demographic characteristics in pediatric EEG papers. Our findings are largely in line with previous reports of other developmental domains across journals (Singh et al., 2023). Similarly, we observe a similar distribution of data collection across different countries as to those seen in other neuroscientific reports in adulthood (Duncan and Rae, 2024). Although far from acceptable, the reporting rates we observed across all demographic measures are considerably better than similar analyses of EEG and fMRI in adults, which showed lower sample sizes and worse reporting of key demographic information (Clayson et al., 2019, Gard et al., 2024, Goldfarb and Brown, 2022). One reason for the need for larger sample sizes in pediatric studies may be the increased noise in EEG data from motor artifact and protocol compliance as compared to adult studies. Moreover, the differences between journals highlight that most studies may have collected most of this information (especially if conducted in the US and was federally funded) but failed to report it in their manuscripts.

A first step towards improving reporting would be to implement mandates of reporting and incorporate standardized reporting guidelines across scientific journals to the extent that it is possible. For example, several developmental journals have recently begun asking or requiring authors to report basic demographic information of participants, including race, ethnicity, and information on the geographical context of the study. These journals include *Child Development* and *Developmental Science*. For other journals, although steps have been taken to ensure that researchers are reporting some demographic data (e.g., sex and gender), several of the top academic journals in developmental EEG (e.g., *Developmental Cognitive Neuroscience*, *PLOS One,* and *Scientific Reports)* have yet to require authors to provide other demographic information (e.g., race, ethnicity, SES, and geographical location). This is reflected in the results from the current review, as sex/gender were almost universally reported, and *Child Development* consistently had higher reporting of demographic information compared to other journals, suggesting these mandates might be successful. Singh et al. (2023) also reported initial evidence that mandates are effective, such that journals that instituted mandates in 2022 (e.g., *Child Development*) observed an increase in the reporting of race, ethnicity, and geographic location that year compared to previous years. However, this was not true of all journals with mandates. Given that these mandates are relatively recent, follow-up reviews of demographic reporting will be needed to evaluate the effectiveness of these mandates in the long term.

Even if these mandates are successful, there is still an open question regarding what specific information authors should report and how it should be reported. In this review, we focused on race, ethnicity, sex/gender, SES, and geographic location of recruitment, as they are considered the most rudimentary information to contextualize the sample and evaluate the generalizability of the findings, especially in Western samples. However, it is important to note that these demographic measures are based on the US Census (US Census Bureau, 2024) and are not appropriate in all contexts. For instance, questions of race and ethnicity are not considered standard, or even appropriate, in some countries and questions about nationality might be more common. This is reflected in the results of this review, such that studies conducted in the US were more likely to report race and ethnicity, compared to studies conducted in other countries. These findings highlight the importance of considering global sociocultural norms when implementing journal mandates. A recent guideline suggests describing demographic characteristics as appropriate for the region, and when reporting specific demographics is not possible, authors should transparently outline the reasons for not collecting or reporting such sample characteristics (Gatzke-Kopp et al., 2023).

Furthermore, beyond standard demographic characteristics (e.g., race and ethnicity), there are other demographic characteristics that could be asked (and reported) that may better capture children’s lived experiences. For example, other useful demographic data may include information about the participants’ religious background, cultural practices, use of language, and measures of enculturation and acculturation (Singh et al., 2023, Singh et al., 2024). Other alternatives to asking questions about race and ethnicity may include asking participants to report on their identities or aspects of their daily experiences, including experiences of racism and discrimination, as well as perceptions of neighborhood characteristics (La Scala et al., 2023). Because of this, there have been recent efforts to standardize the reporting of demographic information for research with infants and toddlers (Singh et al., 2024). This proposal suggests that reported demographic information should include biological information (e.g., sex), gestational status (e.g., gestational age), health status (e.g., diagnoses), community of descent (e.g., race and ethnicity), caregiving environment (e.g., language spoken), and SES (e.g., income and parental education) whenever possible (Singh et al., 2024). Similar efforts are needed for other age periods and characteristics that might be especially relevant for subdisciplines like developmental cognitive neuroscience, such as handedness, head size, data quality metrics (W. Xu et al., 2024), and information about hairstyling and texture (Adams et al., 2024). Finally, given that these demographic categories change over time and context, another approach is to allow participants to self-report how they identify themselves with as many options as possible and then create categories based on those fine-grained data (Cardenas-Iniguez and Gonzalez, 2024).

### 5.2 Recommendations for increasing representation

Based on the results of this review, it is also clear that developmental EEG research must prioritize improving the representation of our samples. This requires the recruitment of larger and more diverse samples, while balancing practical limitations. There have been several recent calls highlighting this issue across developmental psychology and neuroscience, including other recommendations that outline potential solutions (Choy et al., 2022, Garcini et al., 2022, Gard et al., 2024, La Scala et al., 2023, Singh et al., 2023, Webb et al., 2022). Here, we highlight several notable recommendations that are particularly relevant to developmental EEG.

**Increasing Representation of Developmental EEG:** The main recommendation to increase representation in developmental neuroscience, while ensuring ethical and beneficial outcomes for both scientific research and participants, is to engage with the community as partners throughout the research process (La Scala et al., 2023). By considering the perspectives of both researchers and participants, these partnerships help align scientific efforts with the interests and perspectives of the communities being studied. One way to do this is by taking a Community-Based Participatory Research approach (CBPR; La Scala et al., 2023). In CBPR, researchers work in close collaboration with members of the community, who help guide the research process, and play an active role in the development and modification of the research methods and procedures. The main objective of this approach is to give participants a voice and to gain the trust of populations who have largely been excluded from or even harmed by research in the past. This collaborative approach can help reduce biased assumptions about generalizability, enhance the inclusiveness of EEG research, and improve the interpretation and contextualization of findings by incorporating participants' lived experiences. In this way, CBPR can significantly enhance our understanding of how different demographic characteristics influence both the conduct and findings of developmental EEG research, ultimately increasing its representation.

Although this approach has been used in other fields related to sociology, it is rather new to developmental neuroscience. There are several notable examples highlighting the success of this approach (Foster et al., 2024, La Scala et al., 2023, Troller-Renfree et al., 2022). For more detail on this approach, La Scala et al. (2023) recently provided an excellent guide and example of CBPR in practice for developmental neuroscience. These partner-based and community-engaged research approaches hold great promise to increase the diversity and representation of developmental neuroscience research, as well as to ensure studies are conducted in an ethical and accurate manner.

For EEG in particular, there are concerns about a lack of representation for participants in some racial and ethnic groups because of specific hair types and styles that can hinder EEG data collection (Adams et al., 2024, Choy et al., 2022). As indicated in the results of this review, these groups are underrepresented in developmental EEG. This could be because of the composition of the initial samples recruited (e.g., exclusion criteria), as well as specific individuals who are excluded from the studies due to poor data quality that results from current limitations in standard EEG recording procedures (Adams et al., 2024, Bradford et al., 2024, Webb et al., 2022). There are several recently proposed solutions, including working with bioengineers to develop more inclusive EEG systems designed for use with diverse hair types (Etienne et al., 2020, Kwasa, 2024, Mlandu et al., 2024). One notable approach involved working with the company that develops and sells one of the most commonly used EEG systems for developmental EEG to increase the length of the EEG pedestals. This change led to better contact with the scalp and better data quality in infants with curly or tightly coiled hair in South Africa (Mlandu et al., 2024).

Other approaches have focused on providing recommendations and materials to increase the quality of the EEG data, as well as the inclusion, comfort, and trust of underrepresented participants (Adams et al., 2024, Lees et al., 2024). Notably, these recommendations were created in collaboration with community members and researchers who belong to those communities (Adams et al., 2024). Beyond practical recommendations for data collection, other recommendations include ways to measure the effects of different hair types to adjust for some of the confounding effects statistically. One recent suggestion is to include the amount of gel used while collecting EEG as a covariate, which reduces, and in some cases, eliminates potential differences between hair types in data quality (Lees et al., 2024). In summary, by creating more inclusive EEG systems and improving how these systems are used, these approaches will foster more inclusive and representative samples in developmental EEG studies. Future reviews can evaluate the success of these approaches in making developmental EEG more diverse and representative.

**Increasing Sample Sizes of Developmental EEG Studies:** Another notable finding of this review is the relatively small sample sizes commonly employed in developmental EEG studies, suggesting that studies are adequately powered to detect medium-to-large effect sizes for between-subject analyses and medium effect sizes for within-subject analyses. Notably, the results of this review suggest that the sample sizes of developmental EEG studies have increased in recent years. Developmental and neuroscience studies have historically had relatively small samples (Button et al., 2013, Clayson et al., 2019, Oakes, 2017). This is partly due to the difficulty of collecting infant data and the costs (financial and computational) commonly associated with neuroscientific measures. One potential solution to increase sample sizes includes engaging in large-scale, multi-site collaborative research. There have been several relatively recent multi-site developmental neuroscience studies (e.g., ABCD), including several that have utilized EEG, such as the Autism Biomarkers Consortium for Clinical Trials (ABC-CT) (McPartland et al., 2020) and the HEALthy Brain and Cognitive Development (HBCD) Study (Fox et al., 2024, Volkow et al., 2021). Similarly, there have been efforts to increase replication and improve best practices in developmental (e.g., The ManyBabies Consortium et al., 2020) and EEG studies with adults (e.g., #EEGManyLabs; Pavlov et al., 2021). However, to our knowledge, there are no similar efforts focused on pediatric EEG.

Although these multi-site studies will significantly increase the sample sizes in developmental EEG, they all require significant upfront investment and coordination (e.g., systems, tasks, etc.), which may bring into question the scalability of such projects. Another potential solution to increase sample sizes with less coordination includes other open science practices such as methods and data sharing. These methods allow researchers to combine different datasets for larger analyses, which has been historically difficult due to low consensus in the processing decisions made by research teams (e.g., Troller-Renfree et al., 2024). One way to mitigate this issue is to share raw EEG data, which has been done for other aspects of developmental science (e.g., sharing video data; Databrary; Gilmore et al., 2016) or other neuroimaging modalities like MRI (Bethlehem et al., 2022, Thompson et al., 2020). However, to our knowledge, no repositories of developmental EEG currently exist that would allow similar collaborative and open science efforts.

One final promising solution involves the development of novel technologies that make EEG systems more affordable and accessible for larger-scale, multi-site studies, including in low-income settings. Although EEG is more affordable than other neuroimaging modalities, research- and medical-grade systems currently cost approximately $30,000 to over $100,000 US dollars, with each cap/net costing around $3000 to $4000 dollars. Moreover, these systems are often installed in universities and hospitals in specialized rooms that reduce acoustic and/or electrical noise. This greatly limits who can collect EEG data and who can access these specialized laboratories (e.g., individuals of low SES are less likely to travel), contributing to the lack of representativeness and relatively small sample sizes. However, recent developments in consumer-grade systems have created several options for mobile systems at very accessible prices, ranging from a few hundred to a few thousand US dollars. These systems have been validated and used in EEG research with adults (Badcock et al., 2013, Mathewson et al., 2024, Williams et al., 2021). However, these systems are currently being validated in developmental populations, with initial assessments highlighting the feasibility of using these systems to conduct large-scale investigations at relatively low costs, including in low-income settings (Bhavnani et al., 2022, Troller-Renfree et al., 2021). Future studies comparing with research-grade systems are needed to validate these systems in a variety of settings (e.g., high- and low-income settings) and experimental paradigms, including event-related data. However, these novel EEG systems have the potential to increase where EEG data are collected as well as who is collecting the data, empowering developmental researchers who currently do not have access to neuroscientific methods. Ultimately, diversifying the context and populations being studied is as important as diversifying the researchers conducting the research, as they will determine the questions being investigated.

Finally, it is important that funders consider the importance of generating large and representative samples, including supporting projects that increase the diversity and sample sizes associated with developmental EEG research. Moreover, it is important that funders require the collection of demographic information (where culturally appropriate) and its reporting. Requiring researchers to submit their raw data to public repositories for future analyses that combine data across studies would also be beneficial.

### 5.3 Limitations and future directions

The findings of the current review should be considered in light of several limitations. The current review provides a starting point for evaluating historical and current practices regarding participant recruitment and demographic reporting. Future reviews should be conducted to examine the effectiveness of our recommendations (e.g., editorial mandates), and overall progress in the reporting as well as the recruitment of more diverse samples in developmental EEG. We selected a few journals for this initial evaluation, but future reviews should include other journals, given the differences observed between journals. Future analyses could further examine other factors that influence the reporting as well as the sample composition, such as type of study (e.g., single-laboratory study vs. consortium or multi-site study), study design (e.g., experimental vs. correlational or cross-sectional vs longitudinal), or developmental domains under study (e.g., cognitive vs affective domains). Importantly, the estimates regarding sample composition should be considered rough estimates, given that most studies did not report the sample demographics and, even when they did, demographics were rarely reported in full detail (e.g., only a few categories were reported). Future “mega-analyses” are needed to combine the raw data to provide more accurate estimates of the overall representation of developmental EEG. Finally, future studies should continue to investigate the role of different demographic characteristics on the EEG data collection and task-specific effects, as well as the role that development may play in these relations. This can be achieved by leveraging both large-scale, diverse samples that have broad representation as well as community-engaged research to examine the impacts of demographic factors on the EEG data and how they might vary with age. For example, some hair types or hairstyles may particularly influence data collection at specific ages, cultural values may become especially salient at some ages, or sex effects may emerge during specific developmental periods (e.g., adolescence). Additionally, community-engaged approaches can be utilized to better understand the role of sociocultural factors by incorporating participants' perspectives based on lived experiences.

## 6 Conclusion

The current review provides an initial assessment of previous and current practices in the report of demographic characteristics and the diversity and generalizability of the developmental EEG literature. Results revealed that the majority of EEG studies in six notable journals for publishing pediatric EEG studies do not completely report basic demographic information apart from participant sex/gender. Importantly, these results did not meaningfully change based on the year the study was published, suggesting that this problem has not improved as of 2023. Interestingly, we did find that reporting differed between journals, highlighting a potential solution for the lack of reporting overall. Of the studies that reported race and place of recruitment, samples consisted of mostly White participants from North America and Western Europe. Moreover, the median sample size indicates that most studies are only adequately powered to detect medium-to-large effect sizes. However, sample sizes have increased in recent years. The results align with results from other developmental fields but are considerably better than similar adult EEG studies. Our results and discussion emphasize the need for better reporting of sample characteristics and the recruitment of larger and more diverse samples. We provide initial recommendations to help address the current issues of diversity and representation in developmental EEG studies. Future reviews will be necessary to evaluate progress and if these recommendations are effective. Finally, it is important for developmental neuroscience to diversify who is conducting the research, as that will shape what questions are being asked, highlight potential biases in our measures and reporting, as well as help create solutions to these challenges.

## Funding

Santiago Morales was supported by grants from the 10.13039/100000002National Institutes of Health (UH3 OD023279) and the 10.13039/100000865Bill and Melinda Gates Foundation (INV-047884). George Buzzell was supported by grants from the 10.13039/100000025National Institute of Mental Health of the National Institutes of Health (R01MH131637 and R21MH131928). Sonya Troller-Renfree was supported by a grant from the 10.13039/100000071National Institute of Child Health and Human Development (R00HD104923). Contents of the current report are the sole responsibility of the authors and do not necessarily represent the official views of the funders.

## CRediT authorship contribution statement

**Rodriguez-Sanchez Ramiro:** Writing – review & editing, Data curation. **Nadaya Gina:** Writing – review & editing, Writing – original draft, Conceptualization. **Buzzell George A:** Writing – review & editing, Conceptualization. **Troller-Renfree Sonya:** Writing – review & editing, Conceptualization. **Morales Santiago:** Writing – review & editing, Writing – original draft, Visualization, Supervision, Resources, Project administration, Methodology, Investigation, Formal analysis, Data curation, Conceptualization. **Oh Lauren:** Writing – review & editing, Data curation, Conceptualization. **Cox Kylie:** Writing – review & editing, Data curation.

## Declaration of Competing Interest

The authors declare that they have no known competing financial interests or personal relationships that could have appeared to influence the work reported in this paper.

## Data Availability

Data will be made available on request.

## Footnotes

1 A similar pattern was found when combining race and ethnicity, such that only 31 % reported race/ethnicity.
