# Supplementary material for: Generalizability of developmental EEG: Demographic reporting, representation, and sample size
Source: Dev Cogn Neurosci. 2025 May 13;74:101567. doi: 10.1016/j.dcn.2025.101567 (PMC12150051; doi:10.1016/j.dcn.2025.101567)
Supplement: Supplementary file 1 — Supplementary material [file mmc1.docx]

**Supplemental Information for**

**Diversity and Representation in Developmental EEG: Participant recruitment**

**and reporting**

Santiago Morales^1^, Lauren Oh^1^, Kylie Cox^1^, Ramiro Rodriguez-Sanchez^1,2^, Gina Nadaya^1^, George A. Buzzell^3,4^, and Sonya V. Troller-Renfree^5^

^1^Department of Psychology, University of Southern California, Los Angeles, CA, USA

^2^Department of Psychology, California State University Dominguez Hills, CA, USA

^3^Department of Psychology, Florida International University, Miami, FL, USA

^4^Center for Children and Families, Florida International University, Miami, FL, USA

^5^Department of Human Development, Teachers College, Columbia University, New York, NY, USA

***Corresponding Author**: Santiago Morales (santiago.morales@usc.edu) – 501 Seeley G. Mudd Building University of Southern California, Los Angeles 90089

**Funding:** SM was supported by grants from the National Institutes of Health (UH3 OD023279) and the Bill and Melinda Gates Foundation (INV-047884). GAB was supported by grants from the National Institute of Mental Health of the National Institutes of Health (R01MH131637 and R21MH131928). STR was supported by grants from the National Institute of Child Health and Human Development (R00HD104923). Contents of the current report are the sole responsibility of the authors and do not necessarily represent the official views of the funders.

**Conflict of Interest:** The authors declare no conflicts of interest.

**Search Terms**

The following search terms were used to retrieve the list of studies from PubMed. Separate searches were conducted for each journal, with the final searches completed in January 2024 to include all articles published through 2023.

"(((EEG OR ERP OR Electroencephalography) OR (Electroencephalography[Title/Abstract] OR EEG[Title/Abstract] OR ERP[Title/Abstract] OR (event-related[Title/Abstract] AND potential[Title/Abstract]))) AND English[Language] AND Journal Article[ptyp] AND Humans[Mesh] AND (infan*[tiab] OR child*[tiab] OR adolescent*[tiab] OR teen*[tiab] OR pediatrics[tiab] OR baby[tiab] OR babies[tiab])) AND ("Developmental psychobiology"[Journal])) AND (("2011/01/01"[Date - Publication] : "3000"[Date - Publication]))"

"(((EEG OR ERP OR Electroencephalography) OR (Electroencephalography[Title/Abstract] OR EEG[Title/Abstract] OR ERP[Title/Abstract] OR (event-related[Title/Abstract] AND potential[Title/Abstract]))) AND English[Language] AND Journal Article[ptyp] AND Humans[Mesh] AND (infan*[tiab] OR child*[tiab] OR adolescent*[tiab] OR teen*[tiab] OR pediatrics[tiab] OR baby[tiab] OR babies[tiab])) AND ("Scientific Reports"[Journal])) AND (("2011/01/01"[Date - Publication] : "3000"[Date - Publication]))"

"(((EEG OR ERP OR Electroencephalography) OR (Electroencephalography[Title/Abstract] OR EEG[Title/Abstract] OR ERP[Title/Abstract] OR (event-related[Title/Abstract] AND potential[Title/Abstract]))) AND English[Language] AND Journal Article[ptyp] AND Humans[Mesh] AND (infan*[tiab] OR child*[tiab] OR adolescent*[tiab] OR teen*[tiab] OR pediatrics[tiab] OR baby[tiab] OR babies[tiab])) AND ("Plos One"[Journal])) AND (("2011/01/01"[Date - Publication] : "3000"[Date - Publication]))"

"(((EEG OR ERP OR Electroencephalography) OR (Electroencephalography[Title/Abstract] OR EEG[Title/Abstract] OR ERP[Title/Abstract] OR (event-related[Title/Abstract] AND potential[Title/Abstract]))) AND English[Language] AND Journal Article[ptyp] AND Humans[Mesh] AND (infan*[tiab] OR child*[tiab] OR adolescent*[tiab] OR teen*[tiab] OR pediatrics[tiab] OR baby[tiab] OR babies[tiab])) AND ("Child Development"[Journal])) AND (("2011/01/01"[Date - Publication] : "3000"[Date - Publication]))"

"(((EEG OR ERP OR Electroencephalography) OR (Electroencephalography[Title/Abstract] OR EEG[Title/Abstract] OR ERP[Title/Abstract] OR (event-related[Title/Abstract] AND potential[Title/Abstract]))) AND English[Language] AND Journal Article[ptyp] AND Humans[Mesh] AND (infan*[tiab] OR child*[tiab] OR adolescent*[tiab] OR teen*[tiab] OR pediatrics[tiab] OR baby[tiab] OR babies[tiab])) AND ("Developmental Science"[Journal])) AND (("2011/01/01"[Date - Publication] : "3000"[Date - Publication]))"

"(((EEG OR ERP OR Electroencephalography) OR (Electroencephalography[Title/Abstract] OR EEG[Title/Abstract] OR ERP[Title/Abstract] OR (event-related[Title/Abstract] AND potential[Title/Abstract]))) AND English[Language] AND Journal Article[ptyp] AND Humans[Mesh] AND (infan*[tiab] OR child*[tiab] OR adolescent*[tiab] OR teen*[tiab] OR pediatrics[tiab] OR baby[tiab] OR babies[tiab])) AND ("Developmental Cognitive Neuroscience"[Journal])) AND (("2011/01/01"[Date - Publication] : "3000"[Date - Publication]))"
